# What Is the Value of Lived Experience Within Mental Health Professionals, and How Can It Influence Practice? A Systematic Review

**DOI:** 10.1111/inm.70321

**Published:** 2026-08-13

**Authors:** Rebecca Davis, Nicola Clibbens

**Affiliations:** ^1^ Nursing and Midwifery Health Department Northumbria University Newcastle upon Tyne UK

**Keywords:** delivery of health care, international, mental health, public health, social stigma

## Abstract

With one in eight people living with a mental health condition as of 2019, it is reasonable to assume that this population would also include mental health professionals. Despite the global prevalence of lived experience of this nature, its integration into practice and professional roles has yet to be achieved on a wider scale, with its value underestimated and affixed with a significant level of stigma. This study aimed to explore the perceived and actualised value of lived experience among mental health professionals and its influence on practice. A qualitative approach utilising systematic review methodology identified and synthesised the findings of 10 relevant papers. Such an approach acknowledged the range and complexity, and thus the subjectivity, of identified experiences, creating the interpretive space necessary to promote a deeper understanding, from a phenomenological perspective, of how mental health professionals with lived experience utilise their unique knowledge and skills in practice and the value added. Thematic analysis generated two main themes, each with two sub‐themes. These were the *Intrapersonal* and *Interpersonal* value of lived experience in terms of their influence on practice, the former with *Attitudes, Values and Beliefs* and *Deeper Understanding* as sub‐themes; the latter with *Connection* and *Approach to Practice* as sub‐themes. The results of this study have significant implications not only for policy and practice but also for research, calling for social change around mental health stigma. Ultimately, the results highlight the value of integrating lived experience within the healthcare workforce and clinical practice, and the unique perspective and insight it brings.

## 1 Introduction

There is a level of contention surrounding the definition of *lived experience*, specifically, the lived experience of mental health challenges, in terms of the nature and degree of such challenges and the subjectivity of such experiences. For the purpose of this review, a definition used by the *Global Mental Health Peer Network* (GMHPN) was adopted, where lived experience within this context refers to the experience of having, or having had, ‘…difficulties in maintaining mental health as a result of experiencing symptoms of a mental health condition or disorder or psychosocial disability’ (GMHPN 2024, 1). Terminology around the identities and roles associated with lived experience is inconsistent (Byrne et al. 2019), arguably reflecting the complexity and diversity of perspectives surrounding its experience and conceptualisation. Terminology becomes further complicated when the identity and language surrounding *professional* and *patient* merge, in the case of a mental health professional having their own lived experience, creating neologisms such as *prosumer* (professional consumer) (Frese and Davis 1997), *expertience* (portmanteau of expert and experience) (Warne and McAndrew 2007), *lived experience practitioner* (Byrne et al. 2016) and *wounded healer* (Jung 1951).

Those with lived experience and professional mental health care accreditation bring a diversity of experience and knowledge to their practice, as suggested by the concept of *the wounded healer* (Jung 1951). This posits that, through their own suffering, professionals with lived experience are best positioned to understand the suffering of others. It is important to make the distinction that it is not being wounded that facilitates the healing of others, but rather how this experience is accepted, reflected and drawn upon.

International evidence suggests that those employed within caring personal service occupations, that is, ‘caring for children, animals, the sick and the disabled’, are at greater risk of common mental disorders (CMDs) (Stansfeld et al. 2013, 969), also referred to as common mental health conditions (CMHCs), compared to any other occupation. CMHCs include depression, generalised anxiety disorder, panic disorder, phobias, obsessive‐compulsive disorder and CMHC not otherwise specified (Liubertiene et al. 2025). Further to this, the World Health Organisation's (WHO 2022) European Regional Committee found that around one‐third of health and care workers report experiencing symptoms consistent with depression and anxiety (WHO 2025). With this in mind, it is particularly relevant to explore the ways in which lived experience can shape mental health professionals' practice and the value of such. Since its inception, lived experience work has gained traction with the help of the civil rights movements, sustained by factors such as de‐institutionalisation, consumer action and opposition to biomedical theory (Millar et al. 2016).

As well as being directly involved in service provision, lived experience advocates and mental health service staff in designated Lived Experience roles (also known as *peer workers*) also contribute to ‘*education, training, policy design and systemic advocacy*’ (Byrne et al. 2017, 8). Regulatory bodies have introduced guidelines and requirements for involvement that demonstrate a level of acknowledgement of the value of lived experience as observed internationally in countries such as Canada, America, New Zealand and Australia, which, like the United Kingdom, are building a lived‐experience workforce (Byrne et al. 2019). It is important to note that, for the purpose of this review, peer work was excluded as it is an explicitly professionalised role, where lived experience is expected and encouraged to be disclosed and drawn upon, but without the delivery of formal psychological and physiological care or intervention such as that provided by mental health professionals.

As members of the healthcare workforce, professionals with lived experience can also prove invaluable through increased visibility and representation of individuals who identify as mental health professionals who have also experienced/is experiencing mental health challenges and their demonstration of professional competence and success, thereby challenging low expectations, stigma, and discrimination (Hinshaw and Stier 2008). The dispersal of professionals with lived experience within practice could challenge the belief that professionals are ‘exempt from [or] immune to psychiatric struggle’ (Cvetovac and Adame 2017, 348). Increased openness and representation of lived experience among mental health professionals could encourage open dialogue, thereby supporting an ‘upward spiral toward greater visibility’ of professionals with lived experience, rather than a ‘downward spiral toward greater concealment’ (Chaudoir and Fisher 2010, 23).

Roennfeldt and Byrne (2021) state that a lived experience workforce within mental health services, a demographic which mental health professionals are no less likely than the general population to be part of (Harris et al. 2016), has proven invaluable, reducing hospital admission frequency and duration and strengthening the overall social and emotional wellbeing of service users. Mental health professionals openly identifying as part of the lived experience workforce may become a resource to further reduce stigma, as their disclosure could become a form of activism, demonstrating hybrid identities and the continuum of emotional distress (King et al. 2020). Nevertheless, evidence suggests that meaningful collaboration with these individuals is still limited in practice (Byrne et al. 2016; Hennekam et al. 2021); this could be due to a culmination of several factors, including uncertainty and unwillingness to change and hierarchical power dynamics linking to a stigmatic *them and us* dialogue, which contributes to the burden of illness and should therefore be a priority interest of public health (Weiss et al. 2006). Furthermore, not only can the wellbeing of patients be improved through the implementation of lived experience during their delivery of care, but also for the professionals involved in its delivery. For example, concealing part of one's identity due to disclosure‐related distress (Rüsch et al. 2014) can lead to secondary harm due to ‘lost opportunity and personal demoralisation’ (King et al. 2020, 1047). Creating a supportive workplace environment where stigma is challenged, and mental health professionals with lived experience no longer feel the need to conceal this part of their identity, may manage psychological harm (Jones and King 2014). This would further facilitate help‐seeking behaviour (Zaman et al. 2022), which is widely recognised as being limited among mental health professionals (Edwards and Crisp 2017); the delay of which can result in a poorer prognosis for recovery (Clement et al. 2012).

Discriminatory practices in recruitment and employee management have historically reinforced stigma. For example, in 2002, the *NHS* was reported to be habitually avoidant of recruiting professionals with lived experience (Gilbert and Stickley 2012), threatening to violate the Disability Discrimination Act (1995), although this has since been regulated through legislation such as the Equality Act (2010). Furthermore, it is common to request disclosure of previous mental health issues in professional registration licensure to meet fitness‐to‐practise criteria, despite no such request regarding historical physical illness (Peterson 2017). Further, not all individuals who are distressed describe their distress in terms of *mental impairment*, as defined by legislation, potentially making them vulnerable to disability discrimination (Zamir et al. 2022). Overall, the current professional culture within mental healthcare has been summarised as one ‘where stigma is the norm and discrimination or abuses are tolerated’ (National Mental Health Consumer and Carer Forum 2010, 21).

Not only are there significant limitations to current practice and policy, but also to the research and literature informing them. Joyce et al. (2012) noted that literature around professionals with lived experience is often anecdotal, consisting of first‐person accounts. In addition, there is an apparent pessimistic bias within research, focusing on stigmatising experiences and suggesting impaired professionalism, with limited investigation of positive contributions to healthcare (Zamir et al. 2022). Research investigating recovery tends to focus on *clientele* rather than professionals (Cvetovac and Adame 2017), again demonstrating the assumed invulnerability of professionals. Further to this, research around the implementation of lived experience within mental healthcare is said to be under‐theorised and more ideological than empirical (Rowland et al. 2019). This has arguably hindered greater understanding and acknowledgement of the value of lived experience, and therefore its under‐utilisation within practice (Rowland et al. 2019), including that of mental health professionals' lived experience. This ambiguity around the process and value of mental health professionals utilising their lived experience in practice serves to reinforce the existing ‘pervasive culture of nondisclosure’ (King et al. 2020, 1048), as professionals are left uncertain as to how to navigate hybrid identities or what the consequences of this may be.

This paper presents the findings of a systematic review that focused on how lived experience within mental health professionals can positively influence practice and the value of such.

## 2 Method

This review aimed to explore the ways in which mental health professionals with lived experience can shape practice in terms of acknowledgement, acceptance and implementation and the value of such.

### 2.1 Approach and Guidelines

A qualitative systematic review was conducted to understand how mental health professionals' lived experience can influence their practice and those receiving their care, and the value this holds, adopting a methodology that facilitated immersion and familiarity with the data, allowing for a clear yet comprehensive overview to be generated.

### 2.2 Eligibility Criteria

Initial scoping searches of the literature highlighted limited exploration of lived experience within practice and its value within the workplace; therefore, broad eligibility criteria were adopted. Papers eligible for review were required to be available in English and full‐text format. The included literature was limited to a timeframe of 1999 to 2024, as this marked the establishment of NICE guidelines for treatment of high‐quality and ethical standards in 1999 (National Institute for Health and Care Excellence 2013).

Inclusion and exclusion criteria were guided by a predetermined *PEO framework*; *P* (*Population*) = mental health professionals, *E* (*Exposure*) = lived experience of mental illness, *O* (*Outcome*) = influence on practice. Regarding *Population*, papers which focused on peer work were excluded, given that it is an explicitly professionalised role where lived experience is expected to be disclosed and drawn upon appropriately. A decision was made to exclude students with lived experience, as they were deemed to have different roles and responsibilities, and thus different opportunities to implement unique knowledge. Therefore, in terms of inclusion, there was a focus on mental health professional roles, including nurse, psychologist, and therapist.

In terms of *Exposure*, definitions of lived experience varied across the literature; therefore, papers that referenced this experience or relevant key terms were included; this was interpreted broadly to include any experience with mental health, first‐ and second‐hand (vicarious experience as a carer, professional, observer) and current or historical. Factors influencing exposure to mental illness and the occupational psychosocial risks within a healthcare context, such as burnout and moral injury, were considered, particularly the impact of *COVID‐19*. Literature covering this was excluded as it could constitute a direction of enquiry in its own right, given the complexity and severity of its global impact. For example, literature suggested that *COVID‐19* would act as a significant extraneous variable in affecting lived experience of mental illness, as it was found to impact many individuals, including healthcare professionals, as well as placing a severe strain on health services (Muller et al. 2020), which would affect the overall experience of individuals during this period.

Finally, *Outcome* included direct, observable influences on practice, such as conscious decisions to conduct oneself, communicate, or approach care in a particular way in the workplace, as well as implicit influences of lived experience on practice, such as perception of role, professional identity, and health beliefs and concepts. This inclusion enabled consideration of the complexity of professional practice and social interaction within the workplace. Lastly, papers that focused on lived experience implementation in education, training, or research were excluded, given the difference in roles, responsibilities, and expectations compared to those working in a clinical setting.

### 2.3 Search Strategy

Three databases, *CINAHL*, *PubMed* (*MEDLINE*) and *Web of Science*, were selected and searched by a single reviewer in July 2024. *CINAHL* was selected given its focus on nursing and allied health literature, and *PubMed* and *Web of Science* were chosen for their focus within the scientific discipline, specifically biomedical and life sciences.

Pilot searches were conducted using *Google Scholar* to identify keywords and phrases associated with lived experience research to inform database search strings. Considerable variance was identified between terms for those who had lived experience of mental illness and who were also mental health professionals; these included *prosumer* (professional consumer), *consumer‐provider, survivor, expert by experience*, and *wounded healer*. In addition to this, having lived experience was not only shown to include an individual with a mental illness but also their friends, family and carers' experience. Further variation was observed in psychopathological terminology such as mental illness, mental health, psychiatric condition, personal suffering, mental distress, and adversity.

### 2.4 Search Terms

Key search terms were combined with *Boolean* operators (*AND, OR*) into 3 sub‐topics and their derivatives, as informed by the *PEO* structure of the research question. An example database search strategy is displayed in Table 1.

**TABLE 1:** Example database search string (*PubMed*).

Database	Full search strategy
PubMed (Medline)	((“nurse”[Title/Abstract] OR “nurses”[Title/Abstract] OR “nursing”[Title/Abstract] OR “healthcare”[Title/Abstract] OR “mental health professional”[Title/Abstract] OR “mental health professionals” OR “mental health provider”[Title/Abstract] OR “mental health providers”[Title/Abstract] OR “therapist”[Title/Abstract] OR “therapists”[Title/Abstract] OR “counsellor”[Title/Abstract] OR “counsellors”[Title/Abstract] OR “psychologist”[Title/Abstract] OR “psychologists”[Title/Abstract] OR “psychiatrist”[Title/Abstract] OR “psychiatrists”[Title/Abstract] OR “peer” OR “peers”[Title/Abstract] OR “prosumer”[Title/Abstract] OR “prosumers” OR “consumer”[Title/Abstract] OR “consumers” OR “wounded”[Title/Abstract] OR “survivor”[Title/Abstract] OR “survivors”[Title/Abstract]) AND (“lived experience”[tiab:~2] OR “expert experience”[tiab:~2] OR “expertise experience”[tiab:~2] OR “personal experience”[tiab:~2])) AND (“mental illness”[tiab:~2] OR “mental condition”[tiab:~2] OR “mental health”[tiab:~2] OR “mental disorder”[tiab:~2] OR “mentally ill”[tiab:~2] OR “psychological illness”[tiab:~2] OR “psychological condition”[tiab:~2] OR “psychological health”[tiab:~2] OR “psychological disorder”[tiab:~2] OR “psychologically ill”[tiab:~2] OR “psychiatric illness”[tiab:~2] OR “psychiatric condition”[tiab:~2] OR “psychiatric health”[tiab:~2] OR “psychiatric disorder”[tiab:~2])

The full search strategy is displayed in Supporting Information *—*Table S1. Filters were applied to reflect established inclusion and exclusion criteria and narrow search results.

### 2.5 Search Results and Selection Process

There were 2499 initial results (CINAHL, *n* = 774; PubMed, *n* = 1319; Web of Science, *n* = 406), and 2096 after duplicates were removed. All 2096 results were screened by title and abstract to determine eligibility for full‐text review. After initial screening, 50 papers were retained and subject to a second‐phase, more detailed abstract screening and assessment of relevance in terms of which papers could contribute adequate information and insight to cogently address the research question. This refinement was considered necessary due to limited resources. In this second screening step, relevance was determined by the author to be papers that explicitly considered the value of lived experience and its wider impact on practice, hypothesised as a cause‐and‐effect relationship. This second phase of abstract screening was, in part, pragmatic due to the limited resources available and the review being conducted by a single reviewer. After screening, 25 papers were available for full‐text review, which involved assessing all aspects of the literature piece, considering philosophical and methodological positionality and the coherence and implications of findings. Sources for inclusion were determined by their unique characteristics and contextual markers, resulting in a diverse literature base. With informational inclusivity in mind, the author ensured to include first‐person narratives as well as qualitative empirical research.

**FIGURE 1:**
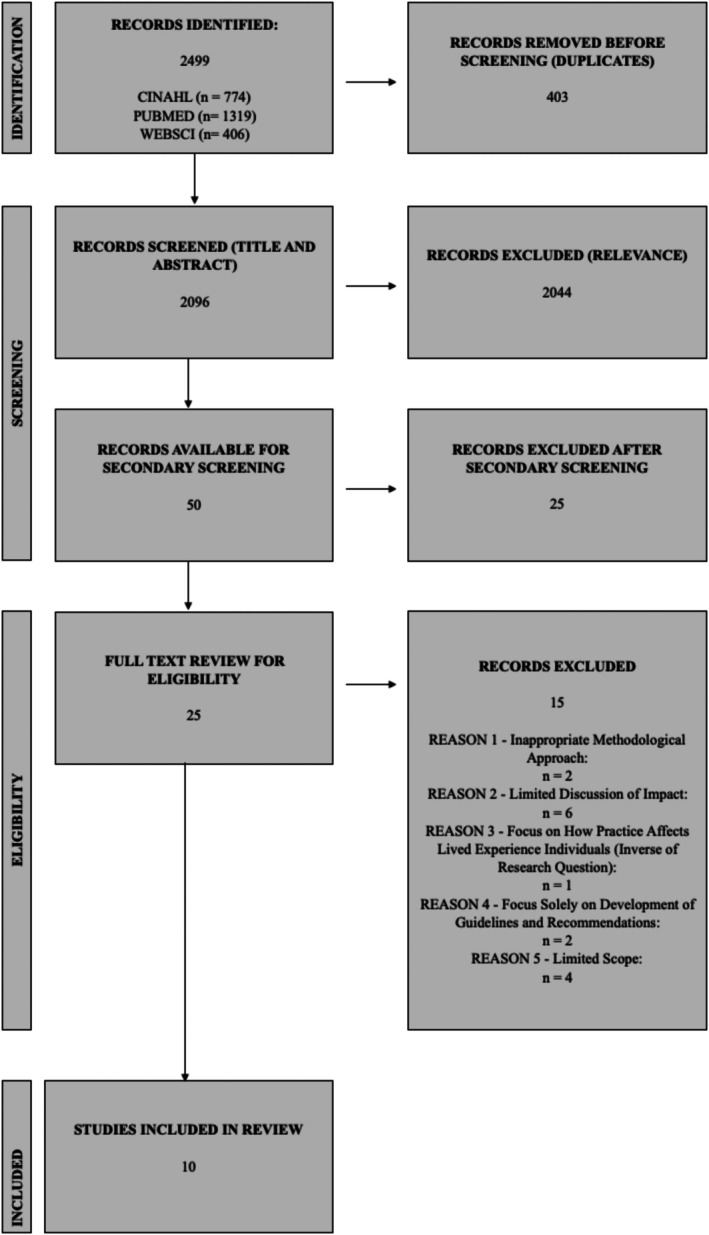
*PRISMA* flow diagram of screening process (Preferred Reporting Items for Systematic Reviews and Meta‐Analysis 2020). Horizontal arrows pointing right represent the outcome of each stage of the screening process Vertical arrows pointing down represent the sequence of each stage of the screening process.

Studies were rejected because of their methodological qualities (*n* = 2), for example, one study used quantitative methods and one was a systematic review; limited consideration of the impact of lived experience on practice (*n* = 6), focus on extraneous factors influencing lived experience implementation within practice, for example, stigma and workplace culture (*n* = 1); focus on guidelines and recommendations (*n* = 2), and limited scope, in terms of autobiographically documenting being a professional with lived experience rather than considering the value of their lived experience and its wider influence within practice (*n* = 4). Eligibility screening resulted in 10 papers for inclusion in the review, as illustrated in Figure 1.

### 2.6 Critical Appraisal

Ten included studies were critically appraised using methodologically relevant criteria and checklists. All papers were qualitative in nature; however, only six were qualitative empirical studies, with the remaining four being first‐person narratives. Therefore, guidance provided by the *Joanna Briggs Institute* (*JBI*) (Aromataris et al. 2015) (Table 2) and the *Critical Appraisal Skills Programme* (*CASP*) (CASP 2023) (Table 3) was first used to assess the methodological quality of the six identified empirical studies.

**TABLE 2:** *JBI* appraisal results.

Citation	(1) Is there congruity between the stated philosophical perspective and the research methodology?	(2) Is there congruity between the research methodology and the research question or objectives?	(3) Is there congruity between the research methodology and the methods used to collect data?	(4) Is there congruity between the research methodology and the representation and analysis of data?	(5) Is there congruity between the research methodology and the interpretation of results?	(6) Is there a statement locating the researcher culturally or theoretically?	(7) Is the influence of the researcher on the research, and vice‐versa, addressed?	(8) Are participants, and their voices, adequately represented?	(9) Is the research ethical according to current criteria or, for recent studies, and is there evidence of ethical approval by an appropriate body?	(10) Do the conclusions drawn in the research report flow from the analysis, or interpretation, of the data?
	Y/N/U/NA	Y/N/U/NA	Y/N/U/NA	Y/N/U/NA	Y/N/U/NA	Y/N/U/NA	Y/N/U/NA	Y/N/U/NA	Y/N/U/NA	Y/N/U/NA
(1) Bhattacharya (2022)	First‐hand Account—Separate Appraisal Needed									
(2) Boomsma‐van Holten et al. (2023)	Y	Y	Y	Y	Y	N	U (Partially)	Y	Y	Y
(3) Cleary and Armour (2022)	Y	Y	Y	Y	Y	Y	Y	Y	Y	Y
(4) Fisher (2023)	First‐hand account—separate appraisal needed									
(5) Karbouniaris et al. (2022)	Y	Y	Y	Y	Y	Y	U (Partially)	Y	Y	Y
(6) Martínez‐Martínez and Sánchez‐Martínez (2023)	First‐hand Account—Separate Appraisal Needed									
(7) Oates et al. (2017)	Y	Y	Y	Y	Y	Y	U (Partially)	Y	Y	Y
(8) Patmore (2020)	First‐hand account—separate appraisal needed									
(9) Richards et al. (2016)	Y	Y	Y	Y	Y	Y	U (Partially)	Y	Y	Y
(10) Waugh et al. (2017)	Y	Y	Y	Y	Y	N	N	Y	Y	Y

**TABLE 3:** *CASP* appraisal results.

Citation	Section A: Are the results valid?						Section B: What are the results?			Section C: Will the results help locally?
	(1) Was there a clear statement of the aims of the research?	(2) Is a qualitative methodology appropriate?	(3) Was the research design appropriate to address the aims of the research?	(4) Was the recruitment strategy appropriate to the aims of the research?	(5) Was the data collected in a way that addressed the research issue?	(6) Has the relationship between researcher and participants been adequately considered?	(7) Have ethical issues been taken into consideration?	(8) Was the data analysis sufficiently rigorous?	(9) Is there a clear statement of findings?	(10) How valuable is the research?
	Y/N/U	Y/N/U	Y/N/U	Y/N/U	Y/N/U	Y/N/U	Y/N/U	Y/N/U	Y/N/U	
(1) Bhattacharya (2022)	First‐hand account—separate appraisal needed									
(2) Boomsma‐van Holten et al. (2023)	Y	Y	Y	Y	Y	Y	Y	Y	Y	(See review for details)
(3) Cleary and Armour (2022)	Y	Y	Y	Y	Y	Y	Y	Y	Y	(See review for details)
(4) Fisher (2023)	First‐hand account—separate appraisal needed									
(5) Karbouniaris et al. (2022)	Y	Y	Y	Y	Y	Y	Y	Y	Y	(See review for details)
(6) Martínez‐Martínez and Sánchez‐Martínez (2023)	First‐hand account—separate appraisal needed									
(7) Oates et al. (2017)	Y	Y	Y	Y	Y	Y	Y	Y	Y	(See review for details)
(8) Patmore (2020)	First‐hand account—separate appraisal needed									
(9) Richards et al. (2016)	Y	Y	Y	U	Y	Y	Y	Y	Y	(See review for details)
(10) Waugh et al. (2017)	Y	Y	Y	Y	Y	N	Y	Y	Y	(See review for details)

All studies demonstrated high methodological quality and thus provided confidence in the evidence using *GRADE CERQual* (GRADE CERQual 2018) as the tool of assessment, as shown in Table 4.

**TABLE 4:** *GRADE CERQual* appraisal results.

Review findings (Themes)	Studies contributing to the review finding	Methodological limitations	Coherence	Adequacy	Relevance	CERQual assessment of confidence in the evidence	Explanation of CERQual assessment
Intrapersonal—attitudes, values and beliefs	1, 2, 3, 4, 5, 7, 8, 9, 10 (9/10 studies)	No: 1, 3, 4, 8 Minor: 2, 5, 7, 9 (Partial explicit consideration of the relationship between the researcher and research process) Moderate: 10 (No explicit consideration of relationship between the researcher and research process and no evidence or statement locating the researcher culturally or theoretically)	No concerns	No concerns	Relevant	High confidence	This finding was graded as high confidence due to few concerns, and a large body of supporting evidence
Intrapersonal—deeper understanding	1, 2, 3, 4, 5, 6, 7, 8, 9, 10 (10/10 studies)	No: 1, 3, 4, 6, 8 Minor: 2, 5, 7, 9 (Partial explicit consideration of the relationship between the researcher and research process) Moderate: 10 (No explicit consideration of relationship between the researcher and research process and no evidence or statement locating the researcher culturally or theoretically)	No concerns	No concerns	Relevant	High confidence	This finding was graded as high confidence due to few concerns, and a large body of supporting evidence
Interpersonal—connection	1, 2, 3, 4, 5, 6, 7, 8 (8/10 studies)	No: 1, 3, 4, 6, 8 Minor: 2, 5, 7 (Partial explicit consideration of the relationship between the researcher and research process)	No concerns	No concerns	Relevant	High confidence	This finding was graded as high confidence due to few concerns, and a large body of supporting evidence
Interpersonal—approach to practice	1, 2, 3, 4, 5, 7, 8, 9 (8/10 studies)	No: 1, 3, 4, 8 Minor: 2, 5, 7, 9 (Partial explicit consideration of the relationship between the researcher and research process)	No concerns	No concerns	Relevant	High confidence	This finding was graded as high confidence due to few concerns, and a large body of supporting evidence

Regarding the four first‐person accounts, those of Bhattacharya (2022), Fisher (2023), Martínez‐Martínez and Sánchez‐Martínez (2023) and Patmore (2020), separate assessment criteria were deemed necessary. There are currently no frameworks or guidelines for assessing the quality of first‐hand accounts. There are, however, recommendations made by several mental health charities for those with lived experience who wish to tell their story (Woods et al. 2022), as well as acknowledged common features within this genre. Most narratives of this kind follow a journey framework when detailing experiences; they are advised to be truthful, grounded in strengths that aid recovery, and to act as “*messages of hope or a ‘call to action*’” (Substance Abuse and Mental Health Service Administration 2017, 25). A flexible approach was adopted during appraisal to avoid invalidating the experience reported by attempting to measure its *worthiness* for inclusion. Instead, there was a focus on whether the text referred to the impact of lived experience and its application in practice.

### 2.7 Data Extraction and Analysis

A spreadsheet was used to record and organise collected information, which was then tabulated (Supporting Information *—*Table S2). Extracted information included year and country of publishing, literature type, study aim/s, participants, method of data collection and analysis, and the reported impact of lived experience. A thematic analysis approach, following Braun and Clarke's (2021) *six‐phase framework*, was adopted to actively engage with the data and identify recurring patterns or themes, as well as the relationships between them. The six phases were conducted as follows: *dataset familiarisation, data coding, initial theme generation, theme development and review, theme refining, defining and naming*, and *reporting* (Braun and Clarke 2021). This approach allowed subjectivity to be used as a resource and for the researcher to become a tool for analysis. In doing so, it was recognised that subjective experiences will have subjective interpretations, reflecting the complex and unique nature of mental health experience.

The initial analysis was inductive, as although initial wider reading of existing literature informed expectations of potential themes, it was deemed necessary to leave interpretative space for unexpected or new themes to be recognised and remain open to participant perspectives, as represented by the author. As well as orientation to data, analysis was shaped by a focus on meaning, which was also evolutionary, starting at a semantic level to establish explicit impacts of lived experience and progressing to a latent level to consider underlying concepts and assumptions of impact. Fluidity in the approach to analysis was important given the social complexity of mental health, in terms of how it is experienced and articulated, considering internal factors such as language and articulation in the *ability* to express, and external factors such as stigma and shame in terms of *freedom* to express. This translational variation between experience and its communication through narratives is emphasised by Mattingly's (1998, 45) claim that there is an ‘anthropological dichotomy between experience as narrated and experience as lived,’ and thus the importance of advancing from a *mimetic position* of interpretation.

The author recognised that they occupy multiple fluid and overlapping identities relevant to the analysis, including academic, health professional and ally to those with lived experience. The author acknowledged the diversity of approaches within the *family of methods* used in thematic analysis (Braun and Clarke 2023), the approach in this review aiming to be reflexive. This approach was chosen as the author acknowledged that whilst it was important to demonstrate an awareness of potential areas of bias, this subjectivity should be used as a tool rather than contained or *bracketed* (Neubauer et al. 2019). Not only would this allow the author to create meaning, but it would also highlight the subjectivity of how lived experience is subjectively presented and subjectively interpreted by others. The author felt ownership of perspectives, both personal and theoretical, would allow them to avoid the *methodolatry* increasingly evident within qualitative research (Chamberlain 2000). Again, given the complexity and subjectivity of lived experience, it was important to acknowledge potential individual variation in the interpretation of selected papers (Nicolson 1995).

Familiarisation with the data set involved reading and re‐reading the 10 selected papers, aiming for full immersion and active engagement with the raw data, making notes of potential patterns. This process of familiarisation led to coding, in which the papers were systematically analysed until a significant level of *information power* (Malterud et al. 2016) was reached, which was determined to be achieved when re‐reading the text produced no further labels with the same analytic focus. Coding software was not used due to limited resources; this process was done manually with the aim of being as comprehensive and inclusive as possible. The concept of *data saturation* was avoided due to its suggested problematic nature (Braun and Clarke 2019). In utilising a reflexive approach, the author therefore joined in ‘rejecting the notion that coding can ever be accurate—as it is an inherently interpretative practice, and meaning is not fixed within data’ (Braun and Clarke 2023, 2).

Initial candidate themes were generated, broadly clustering codes with shared meanings and concepts, and supported by data extracts. When considering themes, the researcher maintained an awareness that *value* could be interpreted subjectively, shaped by their own attitudes, values, and experiences. In generating themes, all categories of value were considered, including value to self, others and the working environments. Stories of struggle and distress paired with silver linings of value added from lived experience of mental illness allowed the author to hold a position of empathy whilst remaining connected to the analysis of the content, humanising the research process and acknowledging stories of vulnerability and authenticity. Mind maps were used to visually represent initial candidate themes, allowing the author to identify those that could be grouped further and merged into a broader theme, or those with significant supporting data could be split into themes or sub‐themes of their own. When conceptualising themes, the author was mindful that themes were not ‘summaries of topics or categories’ but instead ‘captured a core idea or meaning’ (Braun and Clarke 2023, 2). This was ensured by considering whether these themes could have been developed before analysing the data, which the author felt was not the case and that they were interpretations of personal stories.

Themes were reviewed and revised in a recursive process of examination (Braun and Clarke 2021) until it was felt that no further or alternative themes could be generated. This process ensured that themes added value to the analysis outcome by coherently capturing meaningful data centred around a clearly bounded concept. Themes were then refined, defined and named, with each theme serving as a variant articulation of the value of lived experience and its implications for practice. Given the limited scope of this research and the time and resources available, it was crucial to refine the themes to a manageable quantity for synthesis and discussion.

The developed themes were then reported, prioritised in terms of their relevance to the research question and how they could be structured to allow for wider analysis with separate levels of meaning, for example, either breaking them down into sub‐themes, a vertical relationship, or connecting multiple themes with one that is overarching, a horizontal relationship.

## 3 Results

The included studies were qualitative in nature and design, published between 2016 and 2023 and ranged in number of participants from 1 to 27. The studies were produced in several countries, including the United States, the United Kingdom, the Netherlands, Spain and India (Supporting Information *—*Table S2).

### 3.1 Synthesis of Findings

**FIGURE 2:**
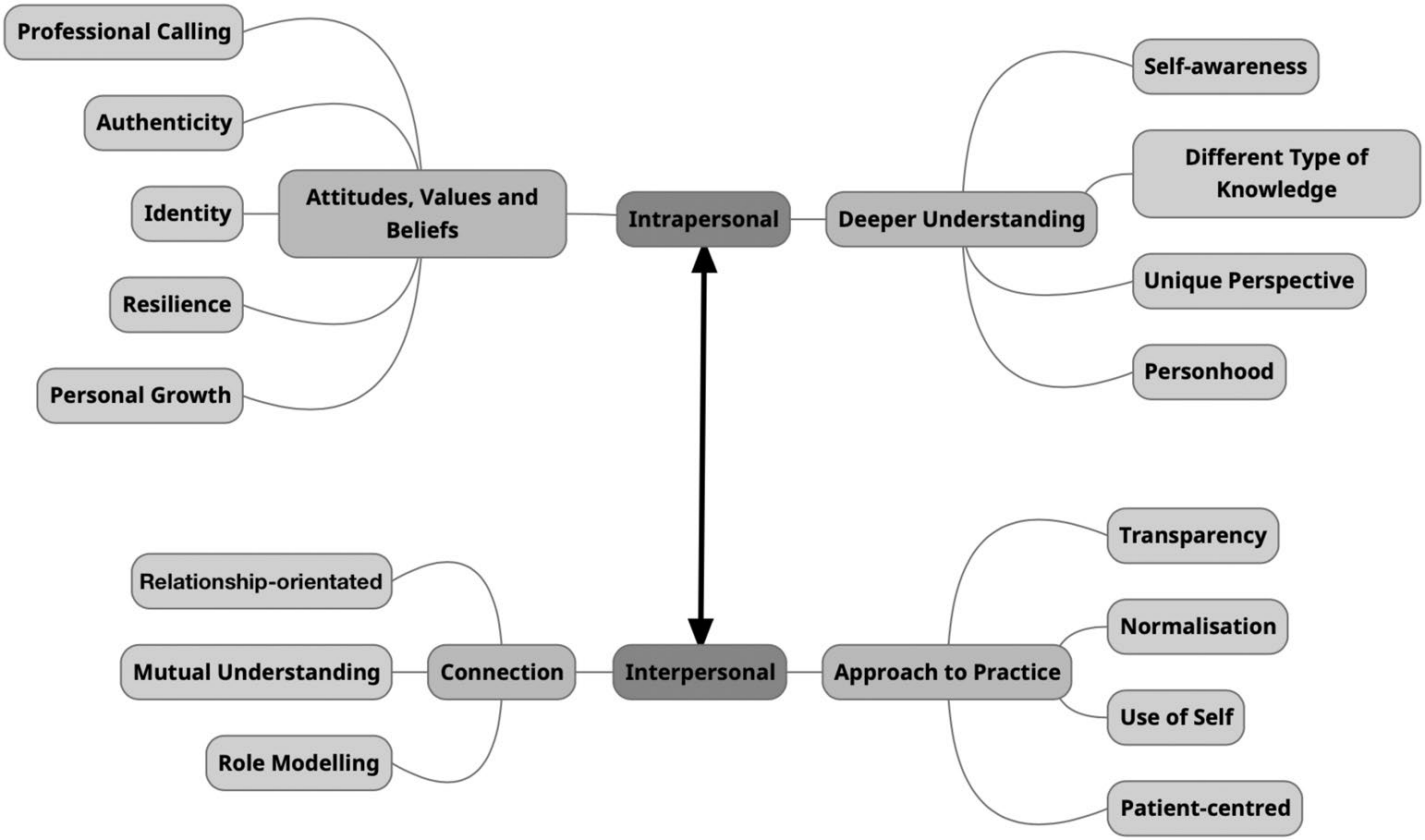
Thematic map displaying themes developed and their relationships. Codes generated in the initial phase of thematic analysis are displayed in outer perimeter of thematic map, connected by curved lines to their related sub‐themes Sub‐themes in lighter grey boxes connected by straight lines to their related main themes in dark grey boxes. Sub‐themes *Attitudes*, *Values, and Beliefs* and *Deeper Understanding* connected to main theme *Intrapersonal* value; and sub‐themes *Connection* and *Approach to Practice connected to Interpersonal* value connected to Double‐headed arrow represents relationship between the main themes of *Intrapersonal* value and *Interpersonal* value.

The analysed data were differentiated and grouped according to how they addressed the research question, as well as mapping links between themes and the influence of their relationship. Overall, two main themes were generated, each with two sub‐themes. The first was the *Intrapersonal* value of lived experience, divided into sub‐themes: *Attitudes, Values and Beliefs*; and *Deeper Understanding*. The second was the *Interpersonal* value of lived experience, divided into sub‐themes: *Connection* and *Approach to Practice*. A thematic map displaying themes and their relationships is shown in Figure 2.

### 3.2 Intrapersonal

#### 3.2.1 Attitudes, Values and Beliefs

A pervasive discourse surrounding the intrapersonal value of lived experience, specifically, its influence on attitudes, values, and beliefs was noted throughout the reviewed literature. The extent to which lived experience shaped perspectives was actively and explicitly touched upon at various levels throughout the literature, as illustrated by Waugh et al. (2017, 3): ‘All participants described a change in their attitudes resulting from their experiences,’ and Bhattacharya (2022, 1): ‘[my mother's] struggle and courage […] have been instrumental in all my personal and professional choices’.

The relationship between lived experience and a professional calling demonstrated how lived experience creates a strong sense of purpose and motivates individuals to align their attitudes, values and beliefs with their chosen profession. For example, ‘People are drawn to mental health because of our personalities and our experiences…’ (Oates et al. 2017, 14). The significance of lived experience in developing a desire to help others through mental health work is that a strong work ethic is built around this motivation (Grabowski et al. 2021). Oates et al. (2017) reinforce the commonality of lived experience as a motivator not only to ‘join[…] the profession’ (p. 8) but also influencing ‘the choice of practice area’ (p. 14), suggesting a level of self‐awareness and acknowledgement of the impact and potential value of lived experience in practice; this links to a long‐standing discourse regarding use of self, known as the *wounded healer* (Jung 1951). There was a suggestion of passivity found within the literature as if the individual were destined to become a mental health professional; this was evident in Bhattacharya's (2022) paper: ‘Psychology chose me because of my mother. She is a survivor …,’ (p. 1) and echoed in Cleary and Armour (2022): … the counsellor had said to me … “*With your life experience, I think [*…*] you would be a good candidate to train in counselling*” (p. 1107).

Another area of transition included personal philosophy, with a focus on resilience and growth. Bhattacharya (2022, 2) stated: “… *my lived experience [*…*] reaffirms my belief in the words of Maya Angelou* (*1994*): *‘*…*still, like dust, I'll rise’*”. Waugh et al. (2017) also demonstrated this perspective, explaining that competency in a professional setting is not lost as a result of lived experience: “many such colleagues were not affected in terms of their ability to do their job” (p. 3); this was supported by Bhattacharya's (2022) personal reflection: “It was reassuring in the sense that I had not lost competencies […] despite my mental illness” (p. 2). In conceptualising resilience and mental health as a journey, Richards et al. (2016) found that a “*personal recovery discourse [*…*] acknowledges ongoing difficulties, but reframes it as a journey rather than a* “*stuck*” *position*” (p. 6).

Integrating lived experience alongside professional knowledge in practice was found to further promote resilience, as it facilitated reflection and healing: “Having this frame of reference may contribute towards acceptance and de‐pathologising […] further facilitating recovery” (Cleary and Armour 2022, 1107). This level of resilience was also found to affect professionals with lived experience's management of their own mental health and wellbeing (Oates et al. 2017). Personal advantages of resilience and growth found within the literature included “feeling proud, stronger, driven, determined, passionate, and valued” (Richards et al. 2016, 10), with professional advantages also described: “I think I'm such a better practitioner for having experienced it” (Oates et al. 2017, 12).

A final way in which lived experience was found to shape attitudes, values and beliefs was through identity evolution and behaviour that was authentic to this, acknowledging that individuals occupy both personal and professional identities, which ultimately influence our behaviour. Boomsma‐van Holten et al. (2023) further reflected this and suggested an overlap between what could be considered two separate entities, personal and professional. A bidirectional relationship between personal and professional identity was also suggested by Cleary and Armour (2022).

In valuing and creating space for lived experience individuals to integrate their identity, “the personal and […] professional identity can further integrate and/or balance, whereby the existing taboo […] can be broken” (Boomsma‐van Holten et al. 2023, 9). This is significant as the perceived dichotomy between professional and patient acts to reinforce barriers for those with lived experience, as illustrated by Fisher (2023, 882): “When the identities, or conceptualisation, of nurse and patient are so seismically different, it poses a huge challenge for someone to embrace both identities at the same time”. The consequences of unintegrated identities are ethical dilemmas (Patmore 2020), identity crises (Fisher 2023) and feeling like an infiltrator (Richards et al. 2016). Given the mounting evidence that an individual can be both a *professional* and a *patient*, conceptions of these roles should be rejected as inaccurate and re‐imagined. Better yet, it should be recognised that these labels are superficial and not a defining feature of an individual; this is reflected by Fisher (2023, 884): “Who am I? That is found within me, not by any role or label. I am more than a nurse. I am more than a mental health patient […] I am. And that is enough”.

#### 3.2.2 Deeper Understanding

A key intrapersonal value of lived experience, as observed throughout the literature, was the development of a *deeper understanding*, not only of others but of oneself. Boomsma‐van Holten et al. (2023) observed how lived experience made individuals “more aware of the spectrum between illness and health,” and “develop[…] a more nuanced view of treatment” (p. 4). This awareness also translated into challenging stereotypes around mental illness (Waugh et al. 2017). As well as a deeper experiential understanding, professionals with lived experience exhibited distinct cognitive processing, or *out‐of‐the‐box thinking* (Karbouniaris et al. 2022). This unique perspective was also found to guide their approach to practice through “provid[ing] a critical view of the rules for communication between professionals and patients” (Martínez‐Martínez and Sánchez‐Martínez 2023, 598).

A common theme within the reviewed literature was that a different type of knowledge is gained from lived experience, which augments theoretical and practical knowledge, enabling greater insight. This does not mean to say that all those with lived experience are *better* professionals, but that they possess a different type of knowledge, which, if utilised, could create a mental health workforce with greater synergy; this line of argument was reflected in texts such as Martínez‐Martínez, Anonymous and Sánchez‐Martínez (Martínez‐Martínez and Sánchez‐Martínez 2023, 598): “Some things are not accessible from the professional's perspective and can only be learnt from the patient's role. The illness can teach things that books cannot.”

Knowledge gained from lived experience was found to allow greater metacognition, acknowledging previous misunderstandings, lack of awareness or gaps in knowledge, as stated in Bhattacharya's (2022) personal account: “I always assumed I understand what my patients undergo”, and “My naivety […]held me back from looking beyond the theoretical concepts of empathy” (p. 1). In a similar vein, a participant in Oates, Drey and Jones' (Oates et al. 2017) study observed that: “Some of the things that I thought would be helpful to people now make me cringe” (p. 12).

Another suggested intrapersonal transformation was a change in perspective, giving individuals “something different to offer” (Fisher 2023, 883). Respondents in Boomsma‐van Holten et al. (2023) study stated that, following their experience, they “realised the relevance of breaking the taboo” (p. 4), suggesting that only once an individual has experienced the stigma that service‐users typically encounter can they truly understand its detrimental impact upon wellbeing and recovery. In appreciating the complexity of mental health and its implications, a more flexible view of recovery could be attained, as shown in Fisher's (2023) paper: “My perspectives on recovery have dramatically altered. Recovery is not an idyllic destination where mental illness is banished […] It is chaotic and complicated […]” (p. 883). Without having experienced significant mental distress, it may be all too easy to place rigid and unrealistic expectations of recovery onto patients, risking the development of a negative self‐concept and feelings of hopelessness. Adding to this, expectations and stereotypes of patient and nurse roles have been challenged following lived experience, as reflected in Fisher (2023): “There is no archetypal nurse or patient. “Patient” *is not an antithesis to* “*nurse*”. *I can use my wounds to help others without becoming a liability*” (p. 884); this also suggests a greater sense of self‐acceptance, asserting that an individual with lived experience can occupy both professional and patient identities.

Linking to the development of unique perspectives from lived experience, narratives of acceptance and appreciation for personhood were observed, as summarised in the words of Fisher (2023, 884): “There is no them and us. There are just people”; this captures the consensus within lived experience narratives that we are all human, and this connects us; therefore, there is no need to “demarcate [and] differentiate,” as we are the product of our own experiences (Richards et al. 2016, 9). This reinforces the idea that an individual can have lived experience *and* also be a mental health professional; there is no separation. The impact of lived experience is clear, reaffirmed by reviewed texts such as Fisher (2023): “My personal experiences […] did not make me a ‘better’ nurse than someone without lived experiences, but it did profoundly impact my nursing practice” (p. 883). Lived experience individuals, therefore, become “*uniquely situated*” (Cleary and Armour 2022, 1108) within professional contributions.

A deeper understanding may lead to personal growth through reflection and the development of self‐awareness. Such elevated awareness and reflection were evident in texts such as Bhattacharya (2022), which reflected: “Despite years of training as a mental health professional, I fell prey to anticipated stigma” (p. 2); this demonstrates the deep‐rootedness of stigma within wider society, as despite training and a level of perceptiveness, stigma prevails. Exhibiting self‐awareness could also influence the practice of others through observational learning, as evidenced by Boomsma‐van Holten et al. (2023) to “[…] initiate a team discussion, in which colleagues become aware of the way they think about patients and the influence of patients on them” (p. 7).

### 3.3 Interpersonal

#### 3.3.1 Connection

In addition to *intrapersonal* value, it was also noted within the literature that lived experience was of significant *interpersonal* value, specifically in developing connections with others and guiding approaches to practice.

A recurring concept of C*onnection* between professional and patient through shared experience and identity was found in the literature; these connections were suggested to develop from a perceived mutual understanding and appreciation for relationship‐orientated care. Through this connection, professionals with lived experience could act as role models, motivating service users and providing hope through their own stories of healing and recovery. Bhattacharya (2022) demonstrated that this connection was not only situational, due to similar experiences:“I was experiencing the same vulnerability [as] my participants” (p. 2), but also extended to an emotional level: “Now, I could empathise emotionally” (p. 2). Connecting through shared experience provoked feelings of proximity and reciprocity (Karbouniaris et al. 2022). This finding serves to promote transparency and acceptance in practice, encouraging professionals to be more open about their lived experience (Boomsma‐van Holten et al. 2023). As a whole, the evidence suggests that uniting people living with mental illness and working in mental healthcare can create a greater sense of community that fosters self‐acceptance, pride and healing (Fisher 2023).

Connection can be facilitated by social behaviours and approaches to practice, such as a professional's relationship‐orientated focus. In valuing the patient‐professional relationship, professionals with lived experience commonly used their experiential knowledge to make “the lines between patient and provider […] less rigid for the purpose of creating a higher degree of therapeutic flexibility and connection” (Patmore 2020, 267). Use of experiential knowledge was either implicit or explicit; though, overt disclosure was seen as a “*judicious act*” only to be used in “pivotal moments in therapeutic relationships” (Oates et al. 2017, 16) and in the patient's best interest to create a “heightened sense of alliance” (Patmore 2020, 266). In fact, Karbouniaris et al. (2022) reported that participants attributed the quality of their relationship with their professional to their decision to disclose lived experience. Through use of self and integrating experiential knowledge in practice, professionals are “able to better understand the ways in which their clients perceive them as well as to gain insight into […] the patient role and the shared, often oscillating dynamics between provider and client” (Patmore 2020, 274).

Incorporated within the patient‐professional connection, a mutual understanding and level of reciprocity indicated that professionals with lived experience felt understood by service users and were able to mutually benefit from the connection through a level of equality and respect, and mutual exchange of knowledge and experience. Whilst healthcare roles, such as nursing, are often considered to be an altruistic profession, it is not beyond the realm of possibility that a mutually beneficial relationship such as this would reinforce professionals' motivation and commitment to nurture it in an act of self‐preservation. Bhattacharya (2022) commented extensively on how they benefited from patient contact and learning from the experiences of people they cared for.

Facilitating professional‐patient connection is the behavioural process of role modelling, as consistently demonstrated throughout the review, expressed as an intentional act or simply as a by‐product of experiential knowledge implementation. Those with lived experience frequently used it as an example for others, demonstrating that recovery is possible: “Bringing my lived experience […] in a therapy session helps many of my patients to understand that one can live satisfactorily and meaningfully despite the presence of symptoms” (Bhattacharya 2022, 2). The credibility of such examples is arguably strengthened by the patient's ability to draw parallels between themselves and the professional with lived experience, as there were fewer perceived differences (Cleary and Armour 2022). Therefore, the professional can act as a realistic example of the possibility of recovery (Cleary and Armour 2022). Professionals used their experience to discuss what had worked for them and help promote motivation for treatment (Boomsma‐van Holten et al. 2023). Professionals with lived experience, through their own transparency and openness, were able to incite self‐acceptance, proposing that “[lived experience] can even be a strength if you dare to be vulnerable” (Boomsma‐van Holten et al. 2023, 6). Not only can the visibility and representation of professionals with lived experience de‐stigmatise lived experience in practice, but it can also address self‐stigma within patients (Karbouniaris et al. 2022).

#### 3.3.2 Approach to Practice

A common area of reflection within the literature was the profound impact and value of lived experience in shaping professionals' approach to practice. The mode of influence was reported to be both “subtle and pervasive” (Oates et al. 2017, 18), but overall, well‐entrenched in all aspects of practice, as lived experience acted as a foundation upon which participants modelled their practice and values (Cleary and Armour 2022). The impact of lived experience guided professionals away from the traditional medical model and towards a more patient‐ and recovery‐centred approach. This transition in professional values could provide an opportunity for “service users to speak where they may have been previously silenced” (Richards et al. 2016, 8). Overall, integrating lived experience and professional identity enables an activist discourse to develop, which challenges current practice and demonstrates allyship. The evidence found within this review is invaluable, as it further asserts the value of lived experience within practice, encourages documentation of these integrated identities to further validate professional competency and draws “these less dominant discourses […] into the foreground, allowing them to be heard more widely” (Richards et al. 2016, 10).

In terms of lived experience manifesting in practice, a general *use of self*, that is, the lived experience aspect of self, was noted throughout the literature. In supporting a patient‐centred focus, experiential knowledge was reported to be a therapeutic tool in professionals' work (Oates et al. 2017). Patient focus can be facilitated by the qualities developed through lived experience, such as an appreciation for choice and sensitivity to patient needs (Patmore 2020). Within the literature, professionals were cognisant of the highly subjective and individualised nature of their lived experience, as demonstrated by Boomsma‐van Holten et al.'s (2023, 5): “I can think of what has helped me, but I know that you really have to tailor what fits someone and what they need.” Use of self was repeatedly stated to require great consideration around its purpose and appropriateness, given the potential for therapeutic rupture. This process of careful analysis of use of self was described as complex and diverse (Karbouniaris et al. 2022), requiring time and reflection to learn about how to use lived experience in practice (Boomsma‐van Holten et al. 2023).

On a wider level, use of self can shape practice, “challeng[ing] dominant psychiatric‐medical discourse,” (Cleary and Armour 2022, 1101), providing a “voice and space” for those with lived experience (Bhattacharya 2022, 20). Specific examples of positive social action through use of self include writing personal accounts in professional journals, employing and supporting service user professionals and engaging in relevant research (Richards et al. 2016).

When considering specific acts of use of self and approaches to practice evident in the reviewed texts, a focal point of transparency and normalisation was found. Transparency was acknowledged to be important in building rapport with patients. It was theorised by Patmore (2020) that professionals “are not and cannot present themselves as ‘blank slates’” (p. 267). Not only can transparency evolve relational environments at a micro‐level between patient and professional, but also at a macro‐level within the workplace, creating a culture of receptiveness and acceptance; “Since I'm open, there are a lot of staff and colleagues who are also open to me.” (Boomsma‐van Holten et al. 2023, 7).

Actively concealing lived experience identity and its influence in practice has been described as counterproductive (Karbouniaris et al. 2022), as it reinforces rigid patient‐professional boundaries and perpetuates a sense of secrecy and shame. Normalisation by means of transparency was described by Boomsma‐van Holten et al. (2023) who stated: “It's really becoming more normal […] that you are open about your own mental health,” (p. 7) and Karbouniaris et al. (2022) text stating that transparency allowed patients to see “both the strengths and the vulnerable sides of the professional [and] led to self‐acceptance and a decrease in self‐stigma and shame,” (p. 30). The findings, however, do not suggest that the value added by lived experience makes these professionals *better* than others (Richards et al. 2016).

A particular area of significance is that, throughout the literature, the method and motive of experiential knowledge use were found to determine its value. Many professionals with lived experience actively reflected upon practical elements such as timing, method and dosage. Incorporating lived experience through self‐disclosure, for example, was discouraged if it was solely to act as a personal therapy for the professional to unburden themselves (Boomsma‐van Holten et al. 2023). In terms of the method of delivery, Oates et al. (2017) noted that professionals with lived experience developed their own rules for use of self in practice. These included the use of the third person when self‐disclosing or using “*we rather than ‘I’*; *or ‘you’ or ‘they’*” (Oates et al. 2017, 10). Consideration was noted to have been made around specific clinical settings and patient groups (Boomsma‐van Holten et al. 2023). These include forensic (Oates et al. 2017) and crisis settings, where lived experience use is deemed less suitable due to risk and acuity of illness; as well as patient factors such as age, difficulty with empathy, or resistance and avoidance of professional intervention (Boomsma‐van Holten et al. 2023).

## 4 Discussion

The findings of this systematic review were in line with the hypothesised value of lived experience and the complexity and diversity of its influence on practice. The range of themes further highlights the subjectivity of lived experience and how its influence can vary in terms of manifestation type (behaviour, attitudes, perception, etc.) and significance. Given the evidence gathered, this review supports the line of argument that strengthened professional competence can stem from experiential knowledge and skill. The focus on mental health professionals, with the exclusion of peer work due to its explicit professionalised role, highlighted the level of ambiguity around navigating being both an accredited mental health professional and an individual who has experienced or is currently experiencing mental health challenges. The lack of research or practice guidelines around the dual identity experience in a clinical environment suggests that it may neither be an expected nor accepted circumstance.

As identified in the reviewed texts, professionals with lived experience suggested that the patient‐professional relationship was positively altered by lived experience integration, resulting from the Intrapersonal and Interpersonal development it stimulated; the existing literature emphasises the clinical relevance of this within the therapeutic relationship. For example, Peplau (1952) proposed individualised experience to be central to the creation of a unique blend of *intrapersonal* factors, including ideals, values, integrity, and a commitment to the wellbeing of others. Peplau (1952) theorised that these *intrapersonal* factors are expressed *interpersonally* through self‐presentation and responses to clients. Morse (Morse 1991, 457) suggested that the most significant relationship within healthcare is the *connected relationship*, in which the patient is viewed “first as a person and second as a patient”.

Firstly, the way in which the reviewed texts corroborate the suggestion that lived experience augments intrapersonal factors such as Deeper Understanding and Attitudes, Values and Beliefs aligns with Epistemological perspectives. The main difference between professionals with lived experience and those without lies in the levels of a priori *knowledg*e, which is independent of experience, and a posteriori *knowledge*, which is dependent on experience, as detailed by Williamson (2012). Arguably, all mental health professionals acquire similar a priori knowledge from standardised educational curricula and qualification requirements, but levels of a posteriori knowledge differ significantly when wider life experience is taken into account. This complexity and depth of knowledge are supported by Carper's 1978 theory of *Fundamental Patterns of Knowing*, which segments patterns of knowing into *empirical*, *personal*, *ethical*, and *aesthetic*, the *personal* aspect of which can be linked to knowledge gained from lived experience.

Secondly, the results of this review further support the suggestion that lived experience can influence interpersonal factors such as *Connection* and *Approach to Practic*e. For example, a benefit of lived experience, as shown in this review, is that it can develop traits, skills and understanding which nurture the emotional intelligence required to make such connections. With emotional intelligence comes the ability to empathise with others, central to a helping relationship (Reynolds and Scott 2000). The significance of lived experience in building an empathetic state is that, historically, there has been a noted deficit in the demonstration of patient‐empathy among professionals in the helping professions (Reynolds and Scott 2000).

Interpersonal connection through mutual acknowledgement of lived experience and, thus, shared identities can have a positive impact on the influence of therapeutic relationships, as suggested by Identity Leadership Theory (Haslam et al. 2020). This framework builds on the social identity approach and proposes that mental health professionals, like all other individuals, occupy multiple social identities, both individual and group (Robertson et al. 2025). It could be said that when a professional with lived experience makes this aspect of their identity known to a service user, this can evolve their unique individual identity as “*I*” and “*me*” to a group identity of “*us*” and “*we*”. It is this group membership that creates the conditions for social influence (Turner 1991), and it is the strength of this connection which determines the significance of this influence (Robertson et al. 2025).

It is important to note that the findings of this review do not suggest that the value added by lived experience makes these professionals *better* than others (Richards et al. 2016); this would be counterproductive to normalisation efforts, as it would further promote perceived differences between professionals with lived experience and those without, when in fact, the main goal is for professionals with lived experience to be acknowledged as professional equals.

Given the complexity of mental health experiences and their role in the equally complex and diverse healthcare environment, it is important to consider the external factors that influence the perceived and actualised value of lived experience. For example, the manifestation of the unique knowledge and skills lived experience brings is said to be mediated by aspects of the workplace environment, such as prevailing social, cultural, ethical, economic, legal, and technological factors (Hagerty and Patusky 2003). These theorised external moderators of value were noted during text analysis, although, beyond the scope of the review, the most significant was social stigma. Interestingly, stigma towards professionals with lived experience was observed to extend beyond colleagues, as evidenced by some service users' ostracising behaviour, who perceived these professionals as not *true service users* and were working within a system which has been part of the problem (Fisher 2023). The complexity and nuance of implementing lived experience in practice seem to be exacerbated and sustained by the level of stigma surrounding it.

The development and implementation of clear, comprehensive guidelines for integrating lived experience into practice, as shown in other aspects of practice to mitigate risk and provide transparency (national standards and healthcare policies), have not yet been achieved, despite being the assumed logical method of addressing the current level of uncertainty and lack of clarity. This brings further attention to how the lived experience and mental health of professionals, in general, are tiptoed around. In fact, those taking a proactive stance to navigate this aspect of mental health practice and seeking to conduct self‐relevant research on mental health, also known as *me‐search* (Robertson et al. 2025), are often perceived negatively (Devendorf et al. 2023). Ultimately, this serves to maintain the inaccessibility of clarity around role expectations and utilising lived experience in practice, leaving professionals to navigate this territory unaided and unsupported. It could be argued that the distinct role separation between peer workers and mental health professionals with lived experience in practice only acts to reinforce the stigma and undervaluing of their lived experience, as it creates an organisational climate which suggests one cannot openly be both an individual with lived experience and a mental health professional, as if one identity nullifies the other. This stigma must be addressed for the influence of mental health professionals' lived experience to be fully actualised in practice (Byrne et al. 2019).

## 5 Strengths and Limitations

Available resources limited the review, and given the subject's complexity, a mixed methods approach may have helped build a stronger evidence base with supporting statistical data. Further to these parameters, it was necessary that the studies were screened by a single reviewer, due to the original nature of the review, which was part of an independent postgraduate dissertation.

It could be suggested that using thematic analysis for both the primary datasets (four first‐person accounts) and secondary datasets (six empirical research papers) led to the secondary datasets being under‐considered, as thematic *synthesis* is often used for data of this type. However, given the limited available resources and the timescale for the review, this was deemed a necessary and appropriate decision. Both thematic analysis and synthesis are qualitative methods for identifying, analysing, and reporting patterns/themes within a qualitative dataset. Both methods follow a structured process with similar stages, including line‐by‐line coding, organising codes, and generating analytic and/or descriptive themes. Thomas and Harden (2008) made reference to, and supported, Boyatzi's (Boyatzis 1998, 4) claim that thematic analysis is versatile, and “not another qualitative method but a process that can be used with most, if not all, qualitative methods”, Therefore, this author acknowledges that thematic synthesis may have been a more appropriate approach to identifying patterns within the six secondary datasets, but thematic analysis was still viable and ensured consistency within the parameters of the review.

It may be criticised that the single reviewer did not include all 25 of the relevant papers available for review; rather, they were screened for *relevance*, which is acknowledged to be a subjective judgement; this was justified as an attempt to reflect a range of perspectives and contexts in pursuit of informational inclusivity. The author also noted the limited availability of resources, which would further necessitate refining search results.

It warrants mention that the studies screened in this review did not seek the opinions of service users regarding the value of lived experience within mental health professionals and its influence on practice. This could be viewed as a limitation, however, with existing literature such as that of Audet (2011), Lovell (2017), and Carter and Moran (2025) already focusing on client perspectives, it was perceived by researcher to merit the exploration of the other representative group forming the patient‐professional dynamic, in doing so, the self‐perception of professionals with lived experience and navigation of their dual identities could be explored.

Aspects such as these may be considered a limitation of the review; however, the practice of reflexivity was implemented to further mitigate reviewer bias, including transparent justification of decisions and consideration of how personal perspectives may affect aspects of the study. To advance the level of transparency and reflexivity, along with the use of critical appraisal tools *JBI* and *CASP* to determine the quality of the reviewed literature as detailed in this section, *GRADE CERQual* (Table 4) was also used to assess the risk of bias and overall confidence in the findings. Furthermore, the review was conducted and reported in accordance with *PRISMA* guidelines (Page et al. 2021).

The generalisability of findings was not a major concern in this review, as Braun and Clarke (2021) suggested that research seems to be “deeply embedded in an ideology of statistical and empirical generalisability” (p. 143), which links to an approach of methodolatry (Chamberlain 2000). Given the personal and subjective nature of the collated information, the author perceived it as disingenuous to attempt to discern the generalisability of findings, with the risk of invalidating the unique experiences reported, and overlooking the personhood of such accounts. This attempt to generalise experience may only act to encourage or reinforce stigmatising generalisations about professionals with lived experience. Explicit awareness of context specificity, as shown in this review, allows readers to make their own judgements regarding the implications of findings in a wider context and hold an awareness that, while themes may be identified, they are neither finite nor guaranteed in every experience.

## 6 Conclusion

The results of this review support existing theories advocating for the recognition of the value of lived experience and its integration within practice. This review also brings attention to the stigmatising culture and practices within healthcare and the need to challenge them. New additions to the existing literature, such as this review, are valuable as they provide further insight into a dynamic and multifaceted topic affecting many, from a micro level of personal experience to a macro level of policy implementation and societal norms. This review aspired to provide a refreshing, optimistic view of lived experience and the possibilities for its implementation, while taking barriers and facilitators into account. Practical implications include augmenting existing and informing future professional policies and recruitment initiatives, refining anti‐discriminatory legislation and stimulating research. This review may encourage greater reflection and act as another step towards solidarity between professionals, both with and without lived experience.

Further research would benefit from a focus on *Implementation Science* to disseminate knowledge about the value of lived experience and its influence on practice, and, consequently, to promote the uptake of evidence‐based practices. Currently, there is a paradox between healthcare research and practice: there is a profusion of evidence supporting the value of lived experience, and discussion of the facilitators and barriers to its implementation, yet there has been no significant evidence of progression. Therefore, the next steps would be to “develop and apply implementation strategies that overcome […] barriers and enhance facilitators” (Bauer and Kirchner 2020, 3), as guided by research conforming to the standards of *Implementation Science*, to streamline lived experience incorporation so that it becomes normalised and part of standardised practice. Future research should not only investigate the conceptualisation and implementation of lived experience in practice from healthcare professional and service‐user perspectives, but also at a wider level, targeting healthcare organisations and facilities, the wider community, and the policy environment too. The conductors of this research, as well as their target samples, should be diverse, including “not just clinicians and clinical scientists but also social scientists, economists, systems engineers and health services researchers” (Bauer and Kirchner 2020, 4).

## 7 Relevance for Clinical Practice

This review adds to the collective appeal for workplace transformation through social and structural change to create an environment where mental health professionals with lived experience feel able to connect with and utilise this aspect of their identity and are part of a culture which supports and encourages such. Those without mental illness and thus without the stigma attached to it are arguably in a position of greater privilege and power; therefore, their collaboration as allies is vital in creating social change through collective, sustained action. Further stimulated changes within the workplace could include incorporating mental health professionals' recovery narratives into institutional dialogues as part of a process of *recovery together*, ultimately challenging beliefs about healthcare professionals' mental health, which currently run counter to recovery discourses (Peterson 2017). In doing so, greater balance could be achieved within mental healthcare, and this particularly disempowered and discriminated sector (Byrne et al. 2016) would be able to make a greater impact through meaningful collaboration, creating a workforce with greater synergy.

## Author Contributions

Both authors have made substantial contributions to the submitted work. R.D. led the conception, design, acquisition and interpretation of data, delivery and editing of the work. N.C. supported further text revision and formatting for manuscript development. Both authors are in agreement with the submitted manuscript and meeting the authorship criteria guidelines of International Committee of Medical Journal Editors. Both authors agree to be accountable for all aspects of the work.

## Funding

The authors have nothing to report.

## Disclosure

The authors have not enclosed any identifying information within this document. The authors did not receive any third party payment or services at any time. The authors did not have any association or financial relationship with outside entities. The authors have no patents or copyrights planned, pending, or issued for the intellectual property submitted. There are no other identifiable relationships/conditions/circumstances that present a potential conflict of interest.

## Conflicts of Interest

The authors declare no conflicts of interest.

## Supporting information


**Table S1:** Full database search strategy.

**Table S2:** Data extraction details.

## Data Availability

Additional data that supports the findings of this study are available in the Supporting Information of this article. The original postgraduate dissertation from which this article was developed is available from the author upon request.
